# Modelling *Anopheles gambiae s.s.* Population Dynamics with Temperature- and Age-Dependent Survival

**DOI:** 10.3390/ijerph120605975

**Published:** 2015-05-28

**Authors:** Céline Christiansen-Jucht, Kamil Erguler, Chee Yan Shek, María-Gloria Basáñez, Paul E. Parham

**Affiliations:** 1 Department of Infectious Disease Epidemiology, Faculty of Medicine, School of Public Health, Imperial College London St Mary’s Campus, Norfolk Place, London W2 1PG, UK; E-Mails: chee.shek08@imperial.ac.uk (C.Y.S.); m.basanez@imperial.ac.uk (M.-G.B.); 2 Energy, Environment, and Water Research Center, The Cyprus Institute, Nicosia 2121, Cyprus; E-Mail: k.erguler@cyi.ac.cy; 3 Department of Public Health and Policy, Faculty of Health and Life Sciences, University of Liverpool, Liverpool L69 3GL, UK; E-Mail: Paul.Parham@liverpool.ac.uk

**Keywords:** mathematical modelling, climate change, mosquito population dynamics, *Anopheles gambiae s.s.*, senescence, malaria, vector-borne diseases

## Abstract

Climate change and global warming are emerging as important threats to human health, particularly through the potential increase in vector- and water-borne diseases. Environmental variables are known to affect substantially the population dynamics and abundance of the poikilothermic vectors of disease, but the exact extent of this sensitivity is not well established. Focusing on malaria and its main vector in Africa, *Anopheles gambiae sensu stricto*, we present a set of novel mathematical models of climate-driven mosquito population dynamics motivated by experimental data suggesting that in *An. gambiae*, mortality is temperature and age dependent. We compared the performance of these models to that of a “standard” model ignoring age dependence. We used a longitudinal dataset of vector abundance over 36 months in sub-Saharan Africa for comparison between models that incorporate age dependence and one that does not, and observe that age-dependent models consistently fitted the data better than the reference model. This highlights that including age dependence in the vector component of mosquito-borne disease models may be important to predict more reliably disease transmission dynamics. Further data and studies are needed to enable improved fitting, leading to more accurate and informative model predictions for the *An. gambiae* malaria vector as well as for other disease vectors.

## 1. Introduction

Mathematical modelling is a useful tool for better understanding the epidemiology and transmission dynamics of infectious diseases, including vector-borne diseases (VBDs) such as malaria, and for better targeting and predicting the outcome of elimination efforts [1,2]. Mathematical models allow the integration of complex biological mechanisms (e.g., the lifecycle of the parasite and/or the vector’s life history parameters), the details of which may not be known precisely, into one eloquent representation [3]. Models of VBDs must take into account, either explicitly or implicitly [4,5], the role played by the vectors in disease transmission. Where the vector component is modelled explicitly, it is important that it depicts the vector population dynamics as realistically as possible in the context of the overall biology and ecology of the vector [6,7,8,9].

For mosquito-borne diseases, the advantages of modelling the mosquito stages explicitly are immediately apparent for models aiming to assess the impact of larvicidal or pupacidal interventions [10,11], or for models wishing to ascertain the effect of external influences on each stage of mosquito development [12,13]. This is relevant, for example, when modelling the impact of climate change on VBDs. Climate change and global warming, and the accompanying changes in temperature, rainfall and humidity (leading to desiccation), as well as extreme events, are expected to influence considerably the spread of infectious diseases, particularly vector- and water-borne diseases [14,15,16,17,18,19,20,21]. The transmission of VBDs is sensitive to fluctuations in environmental variables, in large part due to the response of insect vectors to climatic factors: since insects are poikilothermic, their ecology, population dynamics, and spatial distribution depend strongly on weather-related phenomena [22,23,24].

As it has become more widely accepted that climate change and global warming may affect the spread of VBDs, including mosquito-borne diseases, by influencing vector ecology, experimental research has started to define the extent to which vectors’ life-history parameters [25,26,27,28,29] and capacity to transmit diseases [29,30,31,32] depend on climatic variables such as temperature, rainfall and desiccation. Climate is expected particularly to influence vector survival [33,34], one of the key determinants of VBD transmission [35,36,37,38], in addition to development rates, reproduction, behaviour, and feeding patterns [33,39], all of which influence vector population dynamics, seasonal trends and geographic distribution. Given increasing evidence that weather-related factors affect vector life-history traits and hence disease transmission dynamics, it is important to incorporate the role of climatic factors when modelling vector populations and VBDs. Including a temperature component, in particular, is becoming increasingly common in models of vector abundance [9,40,41,42,43,44] and disease spread [45,46,47].

Faithfully modelling the biology and ecology of disease vectors relies upon the availability of comprehensive entomological data to inform model structure and parameterisation [7,48,49,50]. A lack of precise and adequate data may compromise or limit the accuracy and usefulness of the model output if it relies too heavily on the fitting of crucial and relevant parameters [51,52]. Data describing the different entomological processes contributing to vector population dynamics are, therefore, vital to ensure reliable model predictions. In the case of mosquito vectors, current research suggests there is some evidence that mortality is not only temperature dependent, but also age dependent [53,54,55,56]. However, this may only be evident in laboratory settings and it is uncertain whether wild mosquitoes may live long enough for age to become a significant cause of mortality, or indeed whether the reduction in survival due to environmental factors obscures the role of senescence in vector mortality [57,58]. Until recently, most models of VBDs have modelled constant, age-independent vector mortality, but it has been suggested that realistic age-dependent mortality rates may fit observed abundance data better [59,60,61,62,63]. Exactly how to include age-dependent death will depend on the vector species in question, as well as various other parameters connected with the biology of the vector, including feeding patterns and exposure to parasites, pathogens, and insecticides [64].

Despite the accumulation of experimental evidence from different species of disease vectors to support the inclusion of age-dependent survival, many VBD models still rely upon the framework of constant mortality rates, or mortality rates that depend solely on temperature. The study presented here is based upon recent laboratory work demonstrating that *Anopheles gambiae* sensu stricto (hereafter referred to simply as *Anopheles gambiae*), the primary vector of human malaria in Africa, does senesce, and that survival models incorporating age-dependent survival fit mortality data significantly better than those assuming constant, age-independent survival [34]. We present here a set of four mathematical models of *An. gambiae* population dynamics that enable us to test whether the age-dependent mortality observed in experimental data fits abundance data from sub-Saharan Africa better than the standard modelling paradigm of age-independent mortality rates. The models of age-dependent mortality we present here are based on this experimental data advocating that the survival of *Anopheles gambiae* depends on temperature and age, both in the larval and in the adult stages. We also incorporate other recent work on the influence of temperature on the fecundity and hatching rates of *Anopheles gambiae* [65].

This paper is organised in four sections: Section 2 develops the methodology behind model development, parameterisation, and fitting to the mosquito abundance data, as well as the criteria according to which relevant datasets were identified. Section 3 presents the results of a literature search to collate abundance data as well as the results of model fitting, the interpretation of the findings and a critique of our methods and findings. Finally, Section 4 details the key conclusions from this modelling study and places the importance of this work in a wider context.

## 2. Methods

### 2.1. Model Structures and Parameterisation

The lifecycle of the *Anopheles gambiae* mosquito comprises four main stages: eggs, larvae, pupae, and adults. The first three stages are aquatic, and only upon emergence into adults do mosquitoes leave the water. The larval and adult stages are the longest, with the larval phase consisting of four sub-stages (instars), while the egg and pupal stages are typically very short (depending on temperature). It is useful to model each stage explicitly, partly because the development parameters of each stage may vary considerably and the influence of external factors on these parameters may differ markedly, and partly because this enables the modelling of interventions targeted at specific stages (such as larval source management (LSM) for the juveniles or indoor residual spraying (IRS) for the adult females) [11,66]. In the models presented here, each stage of the mosquito lifecycle is modelled explicitly: the four larval instars are grouped into one larval stage (*L*) (due to a lack of instar-specific data), while the egg (*E*), pupal (*P*), and female adult (*A*) stages are all modelled independently. The four state variables (*E*(*t*), *L*(*t*), *P*(*t*) and *A*(*t*)) track the number of mosquitoes within each stage at time *t*.

In order to test whether incorporating age-dependent mortality in *An. gambiae* population models translates into a better fit to observed abundance data, four models were developed. The first model represents a baseline model for comparison, with exponentially-distributed survival times for egg, larval, pupal, and adult stages, and where stage-specific mortality depends only on temperature. In the second model, larval mortality is assumed to be temperature and age dependent, while in the third, adult mortality is modelled as temperature and age dependent. The final model combines age-dependent larval mortality (as in model 2) with age-dependent adult mortality (as in model 3). Egg and pupal mortality is assumed to be age independent in all four models, on the basis that mosquitoes remain in these stages for too short a time to be affected by senescence (and a lack of experimental data to test for evidence to the contrary). The effect of relative humidity (RH) on the survival of *An. gambiae* is not explicitly modelled here, even though there is some evidence that low levels of humidity may influence adult mosquito longevity [67,68].

**Figure 1:**
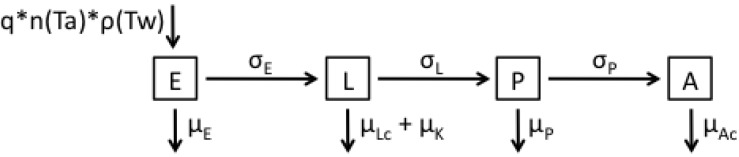
Flow diagram representing the structure of Model 1.

In the following, we describe sequentially the models developed, with Table 1 and Table 2 defining the notation used for model parameters, their values and units, and their sources. Model 1 represents a baseline model (Figure 1 and Equation (1)) that represents survival during the larval and adult stages as dependent solely on temperature, thus assuming that the time spent in each stage is exponentially distributed.

(1) $$\begin{array}{l} \frac{dE}{dt}=qn\left(Ta\right)\text{ρ}\left(Tw\right)A-\left({\text{μ}}_{E}+{\text{σ}}_{E}\right)E,\\ \frac{dL}{dt}={\text{σ}}_{E}E-\left({\text{μ}}_{Lc}+{\text{μ}}_{K}+{\text{σ}}_{L}\right)L,\\ \frac{dP}{dt}={\text{σ}}_{L}L-\left({\text{μ}}_{P}+{\text{σ}}_{P}\right)P,\\ \frac{dA}{dt}=\frac{{\text{σ}}_{P}P}{2}-{\text{μ}}_{Ac}A. \end{array}$$

**Table 1:** Average duration (in days) of egg, larval, and pupal stages at water temperature *Tw* according to Parham *et al.* [69], with corrected coefficients.

Parameter	Functional Form for Average Stage Duration
*d_E_* (eggs)	$\left(1.011+\frac{20.212}{1+{\left(Tw/12.096\right)}^{4.839}}\right)$
*d_L_* (larvae)	$\left(8.13+\frac{13.794}{1+{\left(Tw/20.742\right)}^{8.946}}\right)-\left(1.011+\frac{20.212}{1+{\left(Tw/12.096\right)}^{4.839}}\right)$
*d_P_* (pupae)	$\left(8.56+\frac{20.654}{1+{\left(Tw/19.759\right)}^{6.827}}\right)-\left(8.13+\frac{13.794}{1+{\left(Tw/20.742\right)}^{8.946}}\right)-\left(1.011+\frac{20.212}{1+{\left(Tw/12.096\right)}^{4.839}}\right)$

**Table 2:** Model parameters and parameter values. Parameters marked * were inferred as described below.

Parameter	Definition	Unit	Prior	Posterior
*q*	Proportion of adult females laying eggs	−	0.61 − 0.85 [65]	*
*n*(*T*_a_)	Number of eggs laid per female	−	−1.1057 *Ta^2^* + 56.208 *Ta* – 662.1 [65]	
ρ(*T*_w_)	Proportion of eggs hatching	−	−0.0034 *Tw^2^* + 0.1719 *Tw* – 1.248 [65]	
μ*_E_*	Per capita egg mortality rate	days^−1^	0.32 − 0.8 [10,70]	*
μ*_L_*	Per capita age-dependent larval mortality rate	days^−1^	1/α*_L_*β*_L_*	
μ*_LC_*	Per capita age-independent larval mortality rate	days^−1^	0.0013 *Tw^2^* − 0.0704 *Tw* + 0.9581	
μ*_P_*	Per capita pupal mortality rate	days^−1^	0.25 [11]	
μ*_A_*	Per capita age-dependent adult mortality rate	days^−1^	1/α*_A_*β*_A_*	
μ*_AC_*	Per capita age-independent adult mortality rate	days^−1^	(5.37 × 10^−5^) *e* ^0.228*Ta*^	
μ*_K_*	Per capita density- (and rainfall-) dependent larval mortality rate	days^−1^	${\text{μ}}_{C}\sum_{i=1}^{7}L_{i}/K$ [11]	
μ*_C_*	Constant	days^−1^	0–10,000	*
*K*	Carrying capacity	−	[11]	
τ	Days of rainfall contributing to carrying capacity	days	<10 [11]	*
α*_L_*	Shape parameter of larval gamma hazard function	−	
β*_L_*	Scale parameter of larval gamma hazard function	days	−0.0112 *Tw^2^* + 6.0775 *Tw* – 6.709	
α*_A_*	Shape parameter of adult gamma hazard function	−	3	
β*_A_*	Scale parameter of adult gamma hazard function	days	171.26 *e*^−0.1191*Ta*^	
σ*_E_*	Per capita egg development rate	days^−1^	1/*d_E_* [68]	
σ*_L_*	Per capita larval development rate	days^−1^	1/*d_L_* [68]	
σ*_P_*	Per capita pupal development rate	days^−1^	1/*d_P_* [68]	
σ*_A_*	Per capita adult development rate	days^−1^	1/*d_A_* [68]	
Δ*_T_*	Difference between environmental air and water temperature	°C	2.9 − 7.6 [71]	*

**Figure 2:**
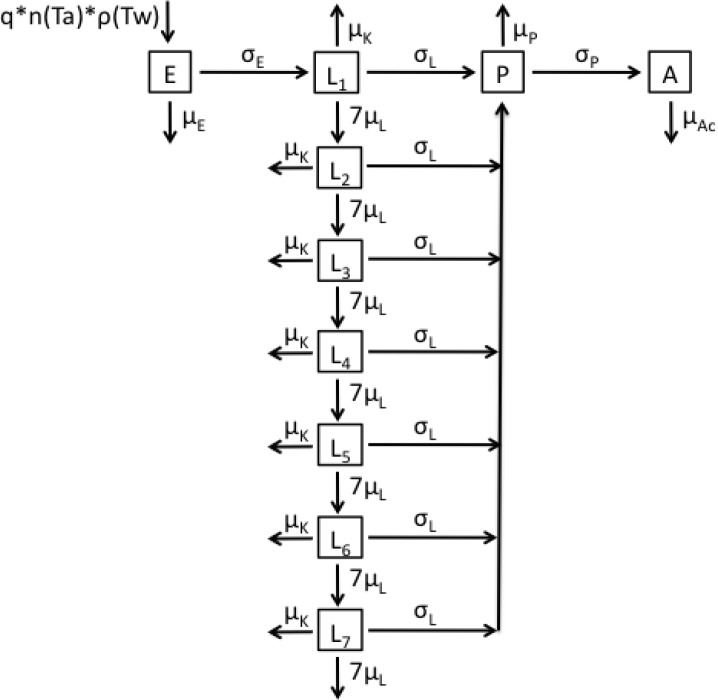
Flow diagram representing the structure of Model 2.

The second model (Figure 2 and Equation (2)) takes into account age-dependent mortality affecting larvae via a gamma distribution with parameters α*_L_* and β*_L_* by subdividing the larval stage into α*_L_* subclasses (the number of sub-classes is determined as defined below) [72,73]. The rate at which larvae progress through the subclasses is set as α*_L_*μ*_L_*, where μ*_L_* is equal to 1*/*α*_L_*β*_L_*. Upon hatching, eggs will enter the first larval subclass (*L*_1_), in which they either progress to the next subclass (*L*_2_) at temperature-dependent rate 7 μ*_L_*, progress to pupae at temperature-dependent rate σ*_L_*, or die due to overcrowding at density-dependent rate μ*_K_*. This process continues as they progress through all subsequent subclasses.

(2) $$\begin{array}{l} \frac{dE}{dt}=qn\left(Ta\right)\text{ρ}\left(Tw\right)A-\left({\text{μ}}_{E}+{\text{σ}}_{E}\right)E,\\ \frac{dL_{i}}{dt}=\left\{ \begin{array}{l} {\text{σ}}_{E}E-\left(7{\text{μ}}_{L}+{\text{μ}}_{K}+{\text{σ}}_{L}\right)L_{i}\;\;,\;i=1\\ 7{\text{μ}}_{L}L_{i-1}-\left(7{\text{μ}}_{L}+{\text{μ}}_{K}+{\text{σ}}_{L}\right)L_{i}\;,\;2\leq i\leq 7 \end{array}\right.\\ \frac{dP}{dt}={\text{σ}}_{L}\sum_{i=1}^{7}L_{i}-\left({\text{μ}}_{P}+{\text{σ}}_{P}\right)P,\\ \frac{dA}{dt}=\frac{{\text{σ}}_{P}P}{2}-{\text{μ}}_{Ac}A. \end{array}$$

**Figure 3:**
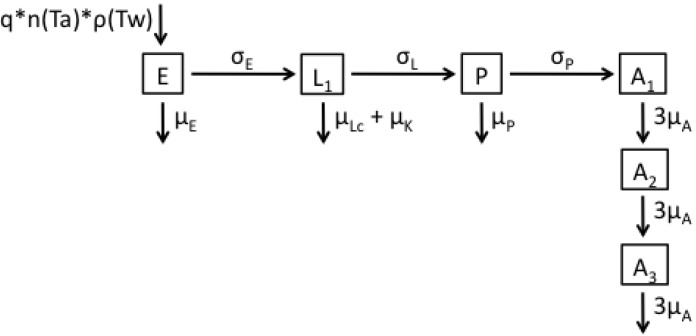
Flow diagram representing the structure of Model 3.

The third model (Figure 3 and Equation (3)) retains the temperature- and age-independent larval mortality of the first model, but now models age-dependent adult mortality. This is modelled in a similar fashion to the age-dependent larval mortality in model 2, namely using a gamma distribution with parameters α*_A_* and β*_A_*. The adult stage of the model comprises α*_A_* subclasses (the number of subclasses is determined as defined below), and adults progress through the subclasses at daily temperature-dependent rate α*_A_*μ*_A_*, with μ*_A_* equal to 1*/*α*_A_*β*_A_*. Upon entering the first adult subclass (*A*_1_), female mosquitoes sequentially progress through adult subclasses at rate 3 μ*_A_*, until they drop out of the model. The number of eggs laid in this model is dependent on the total number of adult females in all three adult subclasses.

(3) $$\begin{array}{l} \frac{dE}{dt}=qn\left(Ta\right)\text{ρ}\left(Tw\right)\sum_{i=1}^{3}A_{i}-\left({\text{μ}}_{E}+{\text{σ}}_{E}\right)E,\\ \frac{dL}{dt}={\text{σ}}_{E}E-\left({\text{μ}}_{Lc}+{\text{μ}}_{K}+{\text{σ}}_{L}\right)L,\\ \frac{dP}{dt}={\text{σ}}_{L}L-\left({\text{μ}}_{P}+{\text{σ}}_{P}\right)P,\\ \frac{dA_{i}}{dt}=\left\{ \begin{array}{l} \frac{{\text{σ}}_{P}P}{2}-3{\text{μ}}_{A}A_{i}\;,\;i=1\\ 3{\text{μ}}_{A}A_{i-1}-3{\text{μ}}_{A}A_{i}\;,\;i=2,3. \end{array}\right. \end{array}$$

**Figure 4:**
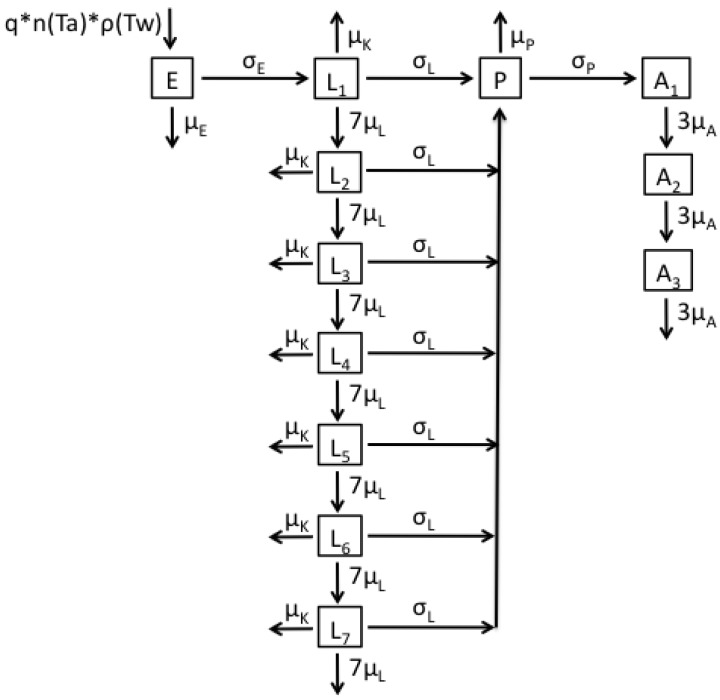
Flow diagram representing the structure of Model 4.

The fourth model (Figure 4 and Equation (4)) is a combination of models 2 and 3, comprising temperature- and age-dependent survival in both the larval and adult stages. The number of eggs laid and the number of pupae developing from larvae are also calculated according to the respective appropriate models.

(4) $$\begin{array}{l} \frac{dE}{dt}=qn\left(Ta\right)\text{ρ}\left(Tw\right)\sum_{i=1}^{7}A_{i}-\left({\text{μ}}_{E}+{\text{σ}}_{E}\right)E,\\ \frac{dL_{i}}{dt}=\left\{ \begin{array}{l} {\text{σ}}_{E}E-\left(7{\text{μ}}_{L}+{\text{μ}}_{K}+{\text{σ}}_{L}\right)L_{i}\;,\;i=1\\ 7{\text{μ}}_{L}L_{i-1}-\left(7{\text{μ}}_{L}+{\text{μ}}_{K}+{\text{σ}}_{L}\right)L_{i}\;,\;2\leq i\leq 7 \end{array}\right.\\ \frac{dP}{dt}={\text{σ}}_{L}\sum_{i=1}^{7}L_{i}-\left({\text{μ}}_{P}+{\text{σ}}_{P}\right)P,\\ \frac{dA_{i}}{dt}=\left\{ \begin{array}{l} \frac{{\text{σ}}_{P}P}{2}-3{\text{μ}}_{A}A_{i}\;,\;i=1\\ 3{\text{μ}}_{A}A_{i-1}-3{\text{μ}}_{A}A_{i}\;,\;i=2,3. \end{array}\right. \end{array}$$

On average, a proportion *q* of female adults will lay a number of eggs *n*(*T_a_*), a proportion ρ(*T_w_*) of which will hatch [65] (where the former depends on the environmental (air) temperature of the adults *T_a_*, and the latter depends on the water temperature *T_w_* in which the eggs are laid. Eggs undergo a fixed, temperature-independent daily mortality at rate μ*_E_*, or progress to larvae at a daily rate σ*_E_*, which is given by the inverse of the duration of the egg stage 1*/d_E_* (Table 1) [69] as defined by Bayoh and Lindsay (unpublished data).

Larvae either undergo a temperature-dependent, but age-independent, daily mortality at rate μ*_Lc_* in models 1 and 3, or a temperature- and age-dependent daily mortality at rate μ*_L_* in models 2 and 4. In addition, larvae are subjected to density-dependent regulation, which is represented by an additional daily mortality rate μ*_K_*. Larvae that do not die progress to pupae at rate σ*_E_*, which is given by the inverse of the duration of the larval stage (Table 1) [69]. Pupae either die at a fixed, temperature-independent daily mortality rate μ*_P_* or progress to adults at rate σ*_P_*, which is given by the inverse of the duration of the pupal stage (Table 1) [69]. Only adult females are explicitly modelled and it is assumed that half of all pupae developing into adults are females [74]. Adults either die at a temperature-dependent, but age-independent, daily mortality rate μ*_Ac_* in models 1 and 2, or at a temperature- and age-dependent daily mortality rate μ*_A_*, in models 3 and 4. The values of fixed parameters in the models are given in Table 2 together with the references of provenance. In the case of the parameters estimated by fitting the models to data (using Bayesian statistics), the prior values are those used as initial values (informed by the literature where available). The asterisks indicate those values that will be obtained from the posterior distribution.

Given experimental evidence that mosquitoes senesce [61,62], the common assumption that their mortality rates are constant may be oversimplified, as it does not take into account that as mosquitoes get older and undergo multiple gonotrophic cycles, they are more likely to die. This is supported by laboratory evidence that survival times are not exponentially distributed, and therefore a constant hazard may not be truly representative of the *An. gambiae* survival function *S*(*t*) [34]. Christiansen-Jucht *et al.* [34] fitted four different parametric forms for *S*(*t*) (exponential, gamma, Weibull and Gompertz) by Maximum Likelihood Estimation (MLE) to laboratory survival data at four different temperatures (23 °C, 27 °C, 31 °C, and 35 °C), and compared the fits using Akaike Information Criterion (AIC). At each temperature, and for both larval and adult stages, the exponential distribution was found to fit the data significantly worse than all other parametric forms, indicating that age-independent survival models are not appropriate to describe *An. gambiae* survival. Although it was shown that the Gompertz distribution fits the experimental survival data best, the gamma function was chosen here since (a) it also fitted the data significantly better than the exponential distribution at all temperatures, and was not significantly worse than the Gompertz in the majority of cases according to the AIC [34]; and (b) the gamma distribution is mathematically and computationally convenient since it can be decomposed into multiple exponentially-distributed models [72].

**Figure 5:**
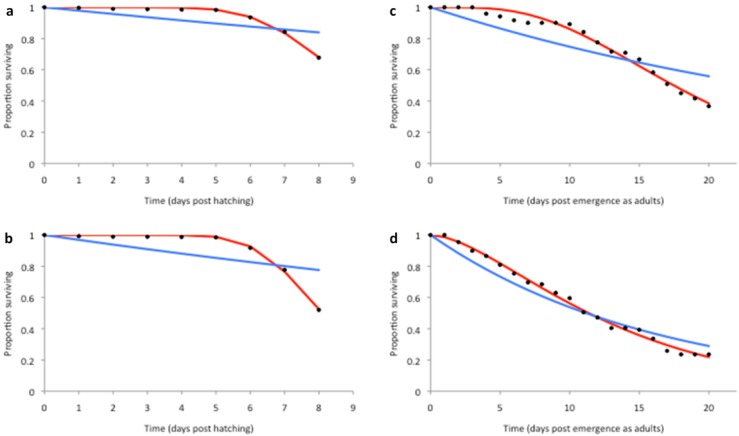
Fit of exponential (blue lines) and gamma (red lines) survival functions *S*(*t*) to the laboratory survival data in [34] at 27 °C (**a**,**c**) and 31 °C (**b**,**d**). Here, (**a**,**b**) are for larvae, while (**c**,**d**) are for adults.

**Figure 6:**
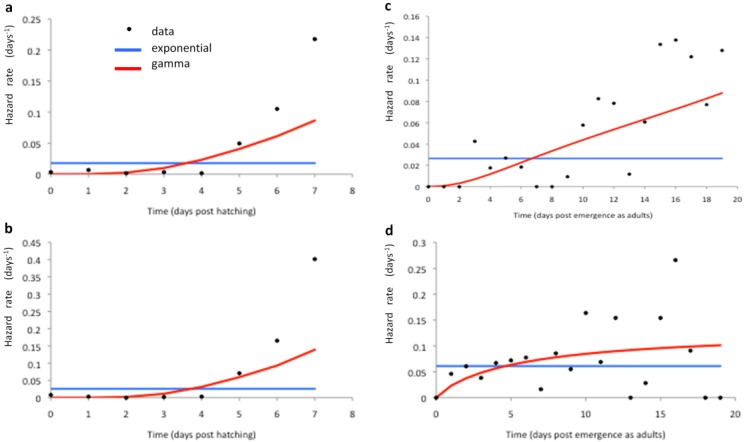
Fit of constant- (blue lines) and time (age) dependent (red lines) hazard functions to the laboratory mortality data in [34] at 27 °C (**a**,**c**) and 31 °C (**b**,**d**). Here, (**a**,**b**) are for larvae, while (**c**,**d**) are for adults. The constant hazard corresponds to the exponential model, whilst the age-dependent hazard corresponds to the gamma distribution of survival times.

**Figure 7:**
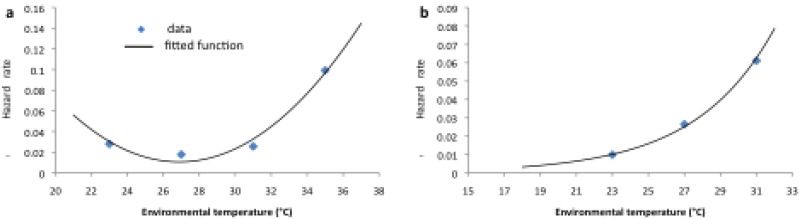
Age-independent larval (**a**) and adult (**b**) mortality rate as a function of environmental temperature (with the best-fit functional forms given in Table 2).

A constant (time-independent) hazard rate, derived from the exponential model fit of *S*(*t*) to the survival data in [34], was fitted to the laboratory mortality data (Figure 5 and Figure 6, blue lines). In addition, the temperature dependence of the hazard functions μ*_Lc_* and μ*_Ac_* was plotted and a functional form fitted (Figure 7 and Table 2) to obtain the larval and adult age-independent mortality rates (μ*_Lc_* and μ*_Ac_* respectively). In order to model more realistically age-dependent mortality, the gamma distribution was fitted to the laboratory survival data in [34] (Figure 5 and Figure 6, red lines).

The gamma distribution is a two-parameter probability distribution; here, both parameters vary with temperature. Since the shape parameter (here, α) corresponds to the number of subclasses into which the relevant stage (larvae or adults) is divided [72,73], α had to be assigned a fixed value independent of temperature. To determine the integer value of α that yielded the closest fit to the MLE-defined best-fit α at the larval and adult stages, we assigned α integer values either side of the original values of α obtained by MLE.

The scale parameter β of the relevant gamma distribution was then obtained by fixing the value of α and fitting β; the goodness-of-fit of the resulting gamma distribution was compared to the original best-fit distribution using AIC. The integer value of α for which the sum of the differences between AIC across all larval or adult temperatures was minimised was chosen as the fixed value of α for that stage (Supplementary Tables S1 and S2). For the larval and adult stages, α was set to seven and three respectively (this is further explained below). The gamma distribution was then fitted by MLE to the survival data with β allowed to vary freely until the best fit was obtained for fixed α.

**Figure 8:**
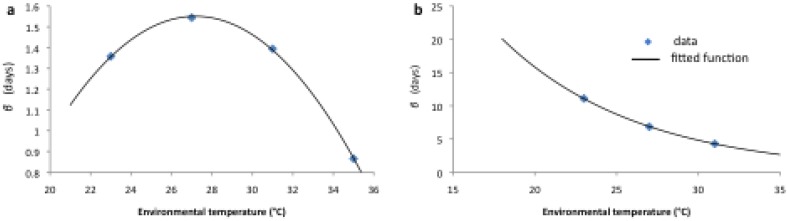
Value of β for the gamma larval (**a**) and adult (**b**) hazard rate as a function of environmental temperature.

The integer values of α and the original best-fit MLE-determined values of α at different temperatures are shown in Supplementary Tables S1 (for the larval stage) and S2 (for the adult stage), along with the difference between the AIC value of the best fit and the AIC values of the different fits for fixed α. The MLE-fitted values of β (for fixed α) were plotted and a functional form fitted to determine how β varies with temperature for each stage (see Figure 8; the fitted functional forms are given in Table 2). To model gamma-distributed survival, while still maintaining a fixed average life expectancy that depends on temperature only, the rate of progression between sub-stages was defined as α*_L_*μ*_L_* for larvae and α*_A_*μ*_A_* for adults, where μ*_L_* = 1*/*α*_L_*β*_L_* and μ*_A_* = 1*/*α*_A_*β*_A_*. This ensures that the average life expectancy of larvae and adults is still 1*/*β*_L_* and 1*/*β*_A_* respectively. As described above, α*_L_* = 7 and α*_A_* = 3, and both β*_L_* and β*_A_* depend on temperature.

In the models presented here, density dependence is only applied to the larval stage, as the mosquitoes are assumed to spend too little time, by comparison, in the egg and pupal stages for density to be a significant limiting factor [75,76]. The additional hazard on larvae to account for density-dependent mortality (μ*_K_*) was derived from White *et al.* [11] as,

(5) $${\text{μ}}_{K(t)}={\text{μ}}_{C}\left(\frac{\sum_{i=1}^{7}L_{i}}{K\left(t\right)}\right),$$

where *K*(*t*) is the environmental carrying capacity at time *t*, and μ*_C_*, which quantifies the magnitude of the density dependence, is a free parameter to be optimised in the model fitting. This formulation assumes the density-dependent mortality rate to be linearly proportional to the total number of mosquitoes in the larval stages. Here, *K*(*t*) is based on the form that was found to fit best the model of [11] to the Garki dataset [77], namely,

(6) K(t)=1τ(1−e−tτ)∫0te−(t−tn)/τrain(tn)dtn.

### 2.2. Longitudinal Data for Model Fitting

A systematic literature review was carried out across the databases PubMed, Web of Knowledge, Google Scholar, and Ovid SP, and all articles published up until 14 February 2015 were considered. Primary key terms *Anopheles*, *gambiae*, *Africa*, *long-term*, *longitudinal*, and *temporal*, and secondary terms *abundance*, *abundance data*, *population*, *density*, *population density*, *mosquitoes*, *monthly*, *weekly*, and *daily* were used in combination with Boolean operators to direct searches. The titles and abstracts of resulting searches were screened for their likelihood to contain data on the collection of *An. gambiae* mosquitoes in any African country over a minimum continuous period of 12 months.

If the initial requirements were met, the full article was retrieved and detailed inclusion and exclusion criteria applied. *An. gambiae s.s.* was the primary focus of data collation, but articles with data on *An. gambiae sensu lato* were also accepted. Records of abundance data collected monthly, weekly, or daily were included provided studies met the requirement of a minimum collection period of 12 months between 1 January 2000 and 31 December 2009. Studies that reported only annual abundance data were excluded. Abundance data for all life-history stages (eggs, larvae, pupae, or adults) were accepted, although the majority of articles reported adult mosquito counts exclusively. Only studies reporting on the natural population dynamics of *An. gambiae* in the wild were included: abundance data collected during implementation of vector control intervention programmes, or from studies that chemically or genetically modified mosquito populations were excluded. Household collection data were accepted only if abundance data were from outdoor collections, and available for all households within the geographical region of the study. The bibliography list of articles meeting all criteria was also examined for additional relevant references.

Where raw data were available, mosquito numbers were lifted directly from the article, while data presented only in figures were extracted using GraphClick, Version 3.0.2 (Arizona Software, 2010). For articles in which abundance data were mentioned (or referred to) but not explicitly given, or where the data could not be recorded, the study authors were contacted directly to request the raw data. When authors did not respond, or if the data did not meet the inclusion criteria, the article was excluded. Where geographical coordinates were explicitly given, the city and country were entered into [78] to retrieve latitude and longitude. Locations were mapped using BatchGeo [79].

### 2.3. Model Fitting

Since the datasets to which model fitting was undertaken reported only the number of mosquitoes caught per month, we assumed that the data (*D*) are independently normally distributed, with a mean equal to the expected average monthly vector abundance for month *i*(δ*_i_*) and a standard deviation σ*_i_* equal to the maximum value of all observations for month *i*. For months with low or no mosquito counts, a minimum standard deviation of 0.1 was assumed. The probability of obtaining the data (*D*), given a model *M* and a set of parameters θ is given by:

(7) $$P\left(D|\text{θ},M\right)=\prod_{i}\frac{1}{\text{σ}\sqrt{2\text{π}}}e^{-\frac{1}{2}{\left(\frac{{\text{δ}}_{i}-n_{F}y_{i}\left(\text{θ},M\right)}{\text{σ}}\right)}^{2}},$$

where *n_F_* is a normalisation factor applied to the simulated data given model *M* and parameters θ (*y_i_*(θ, *M*)) and accounts for any systematic differences between observed abundance and model simulations. This normalisation factor also allows for adjustment between the models, which track the number of adult females, and the data, which include the abundance of male and female adults. Mean monthly mosquito numbers were calculated by averaging across daily model simulation results. All initial conditions were set to unity, and simulations were started six months prior to the date of the first observed data point to reduce the impact of the initial conditions (and model transients) on model fitting.

Assuming a uniform prior for all inferred parameters (marked * in Table 2) within the boundaries specified in Table 2, the posterior probability (the probability of observing θ given *D* and *M*) is defined as,

(8) P(θ|D,M)∝P(D|θ,M).

For each model, we arrived at a set of parameters maximising the posterior distribution, the maximum *a posteriori* (MAP) estimate, through multiple iterations of the basin-hopping algorithm implemented using the in-built SciPy package in Python (v. 2.7). The Bayesian Information Criterion (*BIC*) was calculated to enable inter-model comparisons as,

(9) $$BIC=-2\ln\hat{\text{θ}}+k\ln n,$$

where $\hat{\text{θ}}$ is the MAP estimate, *k* is the number of inferred parameters, and *n* is the number of data points [80]. The commonly used rule of thumb is that models with a difference of ≤2 in BIC values are more or less indistinguishable, and models with a difference of >4 in BIC values are clearly distinguishable [81,82].

We also calculated model probabilities given the posterior distribution around $\hat{\text{θ}}$ Bayesian model selection [83] defines the probability of a model given data, *D*, as,

(10) P(M|D)∝P(D|M)P(M)=P(M)∫P(D|θ,M)P(θ|M)dθ,

where *P*(*M*) is the prior probability of model *M* and *P*(θ|*M*) is the prior probability of parameter set θ. Assuming a uniform distribution for the models and parameters, we have,

(11) P(M|D)∝∫P(D|θ,M)dθ.

By replacing *P*(*D*|θ, *M*) in (11) by its expression in Equation (7), we obtain

(12) $$P\left(M|D\right)\propto \int e^{-\frac{1}{2}{\left(\frac{{\text{δ}}_{i}-n_{F}y_{i}\left(\text{θ},M\right)}{\text{σ}}\right)}_{2}}d\text{θ}.$$

The integral was calculated via Monte Carlo integration with samples from the posterior distribution around $\hat{\text{θ}}$, which were obtained using an adaptive Metropolis-Hastings algorithm [84] with initial conditions in the locality of $\hat{\text{θ}}$. For the sake of simplicity, we assumed that the main contributor to the model probability was the basin of the global optimum represented by $\hat{\text{θ}}$ and discarded the alternative less probable local optima.

For each model, parameter sensitivities explored the impact of simultaneous parameter variations, and were calculated using samples from the posterior distribution around $\hat{\text{θ}}$. We approximated the posterior distribution with a multivariate Gaussian distribution around $\hat{\text{θ}}$, and calculated parameter sensitivities in terms of its covariance [85,86]. The sensitivity of parameter ${\text{θ}}_{i}$ of model *M* was estimated as,

(13) $$S\left({\text{θ}}_{i},M\right)={\text{θ}}_{i}^{2}\Sigma_{ii}^{-1}{|}_{\text{θ}=\hat{\text{θ}}},$$

where ${\text{θ}}_{i}$ is the *i*th parameter of θ and $\Sigma_{ii}^{-1}$ is the inverse of the *i*th diagonal element of the covariance matrix around $\hat{\text{θ}}$. This definition of sensitivity, in combination with the uniform prior, represents a measure of the relative change in model output in response to a relative change in a given parameter value [86].

The climate data adopted were extracted from the ECHAM5/MESSy2 Atmospheric Chemistry (EMAC) general circulation model [87,88,89], in the form of daily average temperatures (t2m) and daily precipitation values. Climatic boundaries were imposed on the model using AMIP-II [90] sea-surface temperature (sst) and sea-ice coverage (sic) assimilation data.

## 3. Results and Discussion

### 3.1. Datasets of An. gambiae Abundance

**Figure 9:**
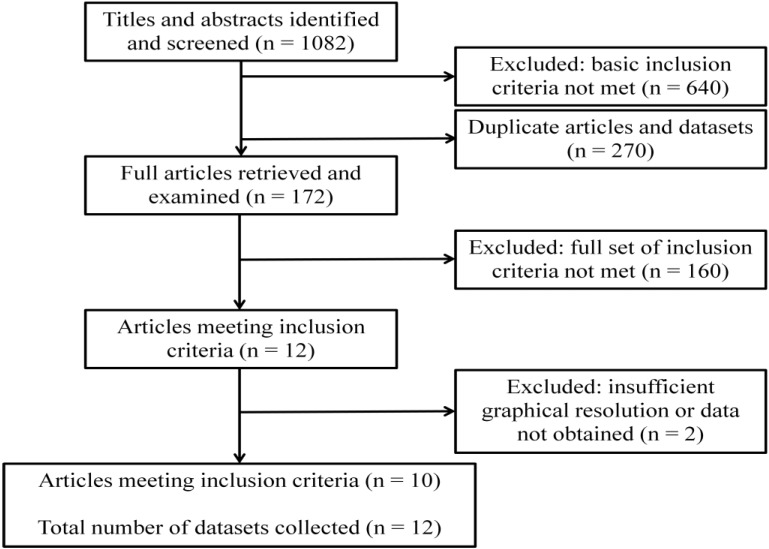
Flowchart of systematic literature review (and results).

**Figure 10:**
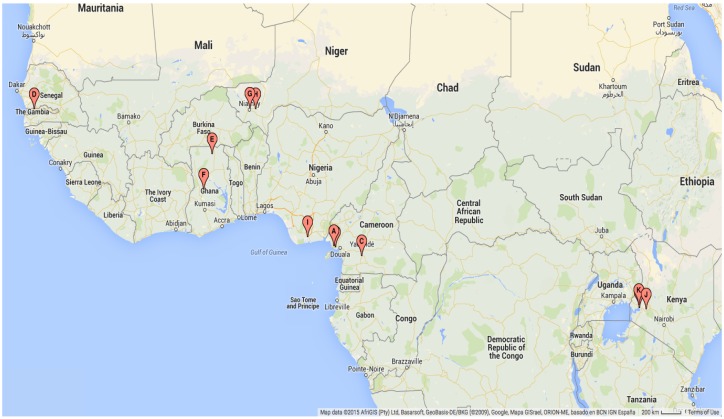
Map of geographical locations of datasets to which the models were fitted.

A summary of the systematic literature review process and results is shown in Figure 9. The review unearthed 12 datasets within 10 publications that fulfilled all inclusion criteria. For each of these datasets, the following information was recorded: title; authors; city, country, and geographical coordinates of the study site; start and end date of the study; study duration in months; and the mosquito species and life-history stage that was counted (Table 3). The mapped locations of the villages or areas across sub-Saharan Africa where the abundance data were recorded are shown in Figure 10.

**Table 3:** Longitudinal datasets of *An. gambiae* abundance.

Map Ref	Geographical Location	Study Dates	Study Duration (Months)	Mosquito Species and Stage
A	Likoko, Cameroon	October 2002–September 2003	12	*An. gambiae s.l.* adults
B	Mutengene, Cameroon [91]	October 2004–September 2005	12	*An. gambiae s.s.* adults
C	Ekombitié, Cameroon [92]	January 2007–December 2007	12	*An. gambiae s.l.* adults
D	Njabakunda, The Gambia [93]	April 2007–March 2009	24	*An. gambiae s.s.* adults
E	Kassena, Ghana [94]	November 2001–October 2004	36	*An. gambiae s.s.* adults
F	Kintampo, Ghana [95]	November 2003–November 2005	25	*An. gambiae s.s.* adults
G	Banizoumbou, Niger [96]	May 2005–December 2006	20	*An. gambiae s.l.* adults
H	Zindarou, Niger [97]	July 2005–December 2006	18	*An. gambiae s.l.* adults
I	Ogbakiri, Nigeria [98]	September 2005–August 2006	12	*An. gambiae s.l.* adults
J	Fort Ternan, Kenya [99]	March 2006–March 2008	25	*An. gambiae s.l.* larvae
K	Lunyerere, Kenya [99]			
L	Nyalenda, Kenya [99]			

Our systematic review indicates that while longitudinal *An. gambiae* abundance data exist for several locations across sub-Saharan Africa at different climates, altitudes, and within different environments, these vary widely in terms of duration, ease of availability, provision of sufficient detail to enable robust model fitting, and overall data quality. Since the main purpose of this review was to assemble a collection of datasets that could be used to inform model fitting, it is clear that longer time-series would enable better model fitting to temporal trends in the data. We found that only half of the datasets obtained described monthly-resolution mosquito abundance for a period longer than 2 years (which would facilitate consistent vector behaviour and climate-driven population response to emerge as distinct temporal patterns, and thus would increase the likelihood of a good fit to the data [100]).

The quality of these datasets, and the level of detail with which abundance data are recorded and/or made available, strongly influenced the ability to fit the four models to these abundance datasets, and, hence, the accuracy of the models’ predictions. Although 12 datasets matched our inclusion criteria, the methods sections in their respective publications almost always reported only the number of mosquitoes caught per month, with very little (or no) information on the number of catches or the frequency of catches within a month. This unavoidably results in model fitting to these datasets requiring *a priori* assumptions in the likelihood expression about the mean and standard deviation around the total monthly number of catches. Here, since the strength (and likely effect) of these assumptions increases for datasets reporting shorter time-series, we fit our four models only to the longest dataset [94].

### 3.2. Model Fitting

**Figure 11:**
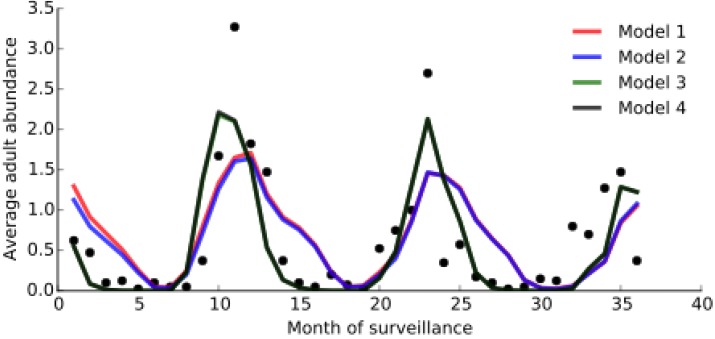
Agreement between data (solid markers) and model predictions (solid lines). Black dots represent the average adult vector counts for each month from November 2001 to November 2004 from [94]. Solid lines show the best-fitting model predictions. Models 1–4 are represented by red, blue, green, and grey lines, respectively.

Figure 11 compares the performance of models 1 to 4 (under their respective best-fit parameters shown in Table 4) with the abundance data reported in [94], and Table 5 gives the BIC values for each model and Pearson’s correlation coefficients (*r*) for the correlation between each model and the data. All four models were consistent in predicting very similar values for the proportion of adult females that lay eggs, and for the daily egg mortality rate. In both cases, the predicted values fell well within the prior ranges predicted by experimental data [10,65,70]. Three of the models, including the two best fitting models (3 and 4), predict a difference between environmental air and water temperature of approximately 7 °C, which is consistent with the higher end of the range observed in experimental data [71], and suggests that models assuming no difference between air and water temperature may not realistically capture the full effect of temperature on survival, likely leading to an underestimate of the influence of temperature on the mortality of the aquatic stages. All models predict very small values for weighting factor on the number of rainfall days *τ* contributing to breeding site carrying capacity (and therefore to density-dependent mortality of the larval stages), although the optimised values of τ given by models 3 and 4 are considerably smaller than those of models 1 and 2. In all cases, this value corresponded to less than one day, which indicates that, for this dataset, the cumulative effects of rainfall are weighted almost equally.

**Table 4:** Fitting-inferred parameter values. Parameters *q*, μ*_E_*, μ*_C_*, τ, and Δ*T* are described in Table 2, and *n_F_* is the normalisation factor applied to the simulated data.

Model	Parameters					
	$n_{F}$	q	${\text{μ}}_{E}$	${\text{μ}}_{C}$	τ	Δ*T*
1	0.466	0.636	0.481	266.915	0.472	3.066
2	0.026	0.613	0.485	16.153	0.689	7.165
3	34.308	0.72	0.501	5886.43	0.087	7.196
4	11.453	0.656	0.533	1753.52	0.095	7.092

**Table 5:** Bayesian Information Criterion (BIC) values for each model, and Pearson correlation coefficient (*r*) values describing the models’ fits to data from [94].

Variables	Model 1	Model 2	Model 3	Model 4
BIC	174.22	174.18	173.82	173.82
*r*	0.65	0.66	0.81	0.81

**Figure 12:**
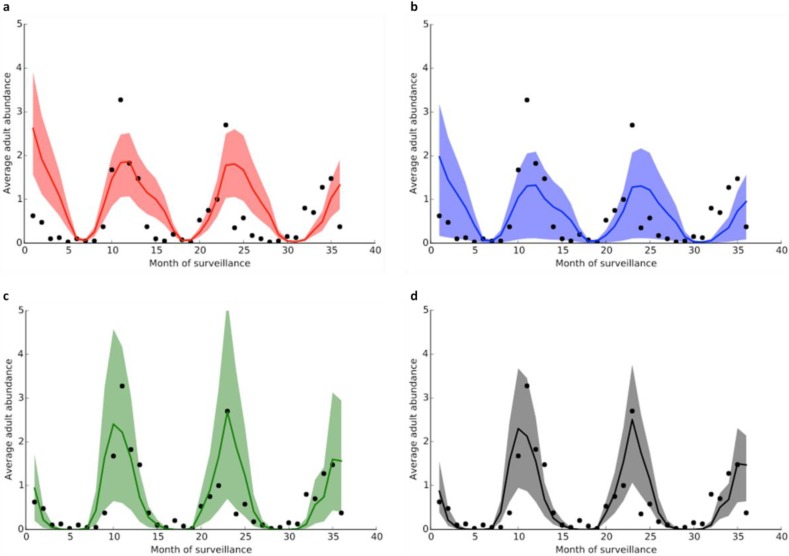
Model predictions (solid colour lines) and 95% Bayesian credible intervals (colour shaded areas) for model 1 (**a**), model 2 (**b**), model 3 (**c**), and model 4 (**d**); black dots are the data and the colour legend is the same as in Figure 11.

The fitted population dynamics and performance of Models 1 and 2 are very similar, and Models 3 and 4 almost identical. The models incorporating adult-only age-dependent mortality (Model 3) and adult and larval age-dependent mortality (Model 4) fit the data better than the “baseline” model with no age-dependent mortality in any stage (Model 1) and with larval-only age-dependent mortality (Model 2). While the difference in BIC values between Models 1 and 2, and Models 3 and 4, is less than 2, information criteria do not provide a measure of statistical significance: models with a lower BIC fit the data better, but there is no measure of how much better they fit [82]. However, the Pearson correlation coefficients show that Models 3 and 4 fit the data considerably better than models 1 and 2, and this is consistent with the 95% credible intervals (CI) shown in Figure 12. It is clear from Figure 12 that the fit of Model 1 is considerably worse than all other models at reliably capturing the main data peaks and troughs, as these points generally lie outside the 95% CI. Model 2 captures the troughs much better than the first model, but the 95% CI still fail to include the extreme peaks in mosquito abundance. The fits of Models 3 and 4 are both much closer to the data and the 95% CI in both models generally capture well both the peaks and troughs in the time-series.

It is evident from the BIC values and correlation coefficients that Models 2 and 4, which include age-dependent mortality in the larval stage, yield no improvement on the fitting compared, respectively, with Models 1 (temperature-dependent mortality only) and 3 (age-dependent mortality in the adult stages only). This suggests that including age-dependent mortality in the larval stages merely serves to increase the complexity and dimensionality of the models, but without contributing to overall model performance or predictive ability. Given the desire, wherever possible, for parsimony in model construction, including age-dependent survival in the adult stages when modelling *An. gambiae* population dynamics appears to be an important requirement for developing more realistic models, whereas including age-dependent survival in the larval stages does not improve model predictions. Given this result, and the fact that age-dependent mortality has been reported in other mosquito species [53,61], it may prove equally important to include senescence in models of other mosquito population dynamics and mosquito-borne diseases. However, the availability of extensive and good quality experimental laboratory and field data on the effects of temperature and age on survival will be necessary to inform such models.

Sensitivity analysis of each model to the six fitted parameters (Supplementary Table S3) indicates that all four models are most sensitive to variations in the proportion of adult females laying eggs (*q*), as well as, to a slightly lesser extent, changes in the daily mortality rate of eggs (μ*_E_*). This is to be expected, as these parameters are crucial in the reproduction, fitness, and survival of the mosquito, and serves to highlight on which entomological parameters we need more detailed and precise data in order to produce more reliable and robust models of disease vectors. All the models are also very sensitive to the difference between environmental air and water temperatures (Δ*T*), which further emphasises the importance of modelling the higher temperatures of the water bodies in which the immature stages of the mosquito develop, and suggests that models assuming water temperatures are the same as air temperatures may be missing out on a subtle determinant of mortality. This also highlights the necessity of measuring water temperature specifically in experimental work that aims to define the effect of temperature on the survival of *An. gambiae* aquatic stages, although this observation is likely applicable to other mosquito disease vectors also. The sensitivity of all models to variations in the difference between air and water temperature also emphasise that the population dynamics of *An. gambiae* are dependent not just on mean environmental temperatures, but also on small temperature fluctuations, which is consistent with previous experimental and theoretical work [9,25]. While models 1, 2, and 4 were robust to variations in the other three inferred parameters, model 3 displayed a greater sensitivity to changes in the values of μ*_C_*, suggesting that mosquito abundance is more sensitive to density-dependent mortality in the larval stages when age-dependent mortality is taken into account in the adult stages only.

In the context of the modelling study undertaken here, two key types of uncertainty should be assessed, namely parameter uncertainty and uncertainty resulting from model structure [101,102,103]. In terms of the latter, we decided to combine all four larval instars into one single stage. This may lead to some imbalance, as there is some experimental evidence that *An. gambiae* may progress through the larval instars at different rates [104], and that there is some heterogeneity (in terms of survival probability, density dependence, and stage duration) between the instars [105,106]. Some existing models assume that the differences between sub-stages are significant, and favour incorporation of larval instars, either explicitly or implicitly, to try and represent this biological heterogeneity more realistically [11,107,108]. The decision not to separate larvae into four instars was based on a lack of experimental data on how temperature and age affect mortality of each instar stage. Further research is, therefore, needed to parameterise models that include instar-specific temperature- and age-dependent survival, although given our findings concerning the apparent greater importance of capturing age-dependent survival of adults compared to larvae, this is arguably less of a priority compared to other current data needs.

The fact that the integer value of α (the shape patameter of the gamma hazard functions) closest to the value obtained by MLE fitting differed by temperature (Supplementary Tables S1 and S2) indicates that the effect of senescence on mosquito mortality may also depend on environmental temperature. In the case where the effect of temperature on senescence is significant, the assumption of a model with a fixed number of larval or adult sub-stages may not be optimal, and a different model structure may be preferable. The models developed here were formulated as ordinary differential equations (ODEs), given that gamma-distributed processes (e.g., survival times) are mathematically convenient and computationally straightforward to express using this type of equations [72]; other waiting time distributions are considerably less convenient, or indeed possible, to model using ODEs. However, there are important limitations to modelling population dynamics using ODEs, in particular the fact that ODE-based models are typically used to represent a population-level framework, not to track individuals. This has implications for the detailed modelling of disease vectors, which may require an individual-based approach to track parity, mating, fertility (and heterogeneities therein), all of which affect vector abundance. However, while the gamma distribution is mathematically convenient to include within this framework, other waiting time distributions are considerably less convenient, or indeed possible, to model using ODEs. Due to these limitations, alternative model structures may, therefore, be more appropriate for modelling temperature-dependent development and mortality, into which the concept of age-dependence may be more readily and naturally incorporated. One such structure is the degree-day (DD) formulation, which is particularly applicable (and widely used) for modelling insects and pests as it models physiological, as opposed to chronological, age and expresses the development of poikilotherms as a function of the difference (in degrees Celsius) between the environment and the minimum temperature for development (MTD) of the insect [109,110,111].

In terms of parameter uncertainty, in addition to the difficulties arising from the limited availability of good quality datasets to enable robust model fitting (discussed in Section 3.1), other assumptions are also unavoidable given current data limitations. The mortality rates of eggs and pupae are assumed to be temperature independent on the basis that both stages are relatively short lived (compared to the larval and adult stages) and may not be significantly affected by external factors (or, if they are, the effect may be comparatively minor to strongly affect abundance prediction). To the best of our knowledge, there is currently no suitable detailed experimental data to suggest that this assumption may be erroneous, but it may be a consideration for future experimental work.

The models developed here assume that the immature and mature stages are entirely independent, but research by Christiansen-Jucht *et al.* [34,65] shows that adult parameters depend on the environmental conditions of the immature stages. While this dependency is ignored in this paper in order to focus on the role and importance of age-dependent mortality in population models of *An. gambiae*, we consider it potentially extremely important and is the subject of work in progress. In addition, since the age-dependent parameters (and some of the temperature-dependent parameters) used here are based on the same work in [34,65], it is important to note that these parameterisations are based on four temperature data points for the larval stages, and three temperature data points for the adult stages, all of which are 3 °C apart. Fitting these parameters to functional forms that reliably hold across a wider range of temperatures of interest is clearly a limitation of model parameterisation, but, as with all parameter uncertainty, is carefully considered through sensitivity analyses. In addition to the limited number of temperatures at which distributions were fitted to the experimental survival data, Christiansen-Jucht *et al.* [34] found that the Gompertz distribution generally represented the best fit. The difference between the Gompertz and gamma distributions’ fits was found in [34] not to be significant for most of the temperature data points considered, and both fitted the survival data significantly better than the exponential distribution (although the Gompertz distribution yielded the best fit overall). In order to incorporate our analysis of the survival data in [34] as realistically as possible, it may be important to model Gompertz-distributed, rather than gamma-distributed, mortality.

The models developed here aim at capturing the population dynamics of *An. gambiae* in the wild, but it should be noted that the calibration of parameters is based on data obtained on laboratory mosquitoes (essentially due to the scarcity of data available on wild mosquito life history parameters). It may be that mosquitoes in the laboratory are not, in fact, truly representative of wild populations, in which case the parameters adopted here may be limited in their application to modelling *An. gambiae* population dynamics in sub-Saharan Africa, and the fit of these models to datasets used should be interpreted with caution. This highlights again the need for better entomological experimental data to inform models. We also note that the parameterisation adopted is time independent, meaning that we assumed there was no phenotypic change between mosquitoes collected in abundance datasets from different periods. However, recent research has suggested that insect vectors can evolve over decadal timescales in response to rapidly changing environmental pressures [112]. This has implications for the common model fitting assumption that different mosquito collection datasets temporally far apart are comparable; the datasets our review unearthed span a period of almost ten years, and stretch across Africa. The models constructed do not account for any spatial and/or temporal heterogeneity in model parameters, nor do they offer a way of testing for such diversity, and this common assumption represents a limitation that also potentially undermines the modelling of other mosquito-disease systems.

Since the models presented here contain a considerable number of parameters, we tried to base as many as possible on existing laboratory or field data to fix them at biologically plausible values in order to contain our analyses and address our research aims as succinctly as possible. It should be noted, however, that all four models are also likely to be sensitive to variations in these parameters that arise due to ongoing limitations in current entomological data quality and quantity; thus, although we have assigned these parameters to the most biologically realistic values (or environmental dependencies) based on the most up-to-date evidence and data on *An. gambiae* life history, further work is undoubtedly required to explore the implications of this additional parameter uncertainty on our findings. In this work, the decision was made to explore only the sensitivity of the models to those parameters that were not based upon experimental data, or for which the range of priors only were based on experimental data, in order to most clearly present our findings and the implications for future modelling of *An. gambiae* (and other mosquito populations for which temperature- and age-dependence has been experimentally shown to exist [34,38,53,54,55,61]). However, as mentioned above, there are undoubtedly considerable gaps in the current entomological data (e.g., life-history parameter dependencies derived from a wide range of environmental variations, heterogeneity between different *An. gambiae* populations due to local adaptations, relatively small sample sizes in the limited number of controlled experiments that have been undertaken, and so on), and therefore the values at which non-fitted parameters were fixed may not be as optimally representative of the mosquito’s biology as we have necessarily assumed. Further sensitivity or uncertainty analyses around these parameters is therefore likely to provide additional useful insight into the extent to which models can rely on current entomological laboratory or field data to inform their predictions and where future experimental and modelling studies are ideally required.

Some of the limitations described here may lead to the effect of senescence being underestimated in our model fits. Yet, despite certain assumptions, the models that include adult-only age-dependent mortality and larval and adult age-dependent mortality still fitted the abundance data better than the other models, and we therefore expect the strength of our result concerning the importance of adult age-dependent mortality to be very conservatively reported here. Reducing the uncertainty around some of the entomological parameters, and using more detailed datasets to inform model fitting and inference, may help to highlight further the true effect and importance of including age-dependent mortality, particularly in the adult stages, in models of vector abundance. We are currently undertaking further research fitting the models presented here and other models to several abundance datasets from across sub-Saharan Africa, to determine whether age-dependent mortality varies between the geographical locations of the mosquito habitats.

Finally, we note that the modelling frameworks developed here are not limited to *An. gambiae s.s.* mosquitoes, or indeed to malaria. While these particular models are only relevant for *Anopheles gambiae s.s.* (by virtue of parameterisation based on entomological data of this species alone), dozens of other *Anopheles* species also transmit malaria [113]. There is currently no detailed experimental evidence to assess whether the life history parameters of one *Anopheles* species may be applicable to others, and this highlights the need for extremely precise experimental data on the many vectors of VBDs. However, provided detailed life-history parameters were available for other species, the framework developed here is readily applicable to other mosquito species to assess the importance and role of temperature- and age-dependent mortality on vector abundance and, ultimately, disease transmission and implications for control.

## 4. Conclusions

Comparison between the models developed here suggests that including temperature- and age-dependent survival in the adult stages of models of *An. gambiae s.s.* population dynamics may provide significantly better fits to longitudinal adult female abundance data from sub-Saharan Africa. Despite the limitations and assumptions of this study, we show that models incorporating age-dependent mortality in the adult stages match temporal trends in observed mosquito data better than models without age-dependent mortality or models that include age-dependent mortality in the larval stages only. Our results indicate almost no difference in model fitting between adult-only age-dependent survival and adult and larval age-dependent survival; thus, another important conclusion of this work is that despite experimental evidence for both larval and adult age-dependent mortality, including age-dependent mortality in the adult stages only represents both the most parsimonious option for modelling and the best fit to data. This suggests that the common assumption of constant adult *An. gambiae* mortality (or even modified to incorporate temperature dependence), or, equivalently, that senescence does not play a large role in driving vector mortality, may be erroneous or, at the very least, a serious underestimation. Nonetheless, this result requires further research and confirmation, both from more detailed vector mortality data collected the field, additional experimental data from the laboratory (e.g., on other mosquito species), and further modelling studies of both the mosquito populations themselves and the diseases they transmit: in the case of malaria and other VBDs, since the inclusion of senescence in models of mosquito abundance appears to enable more accurate and reliable predictions of the population dynamics of a key malaria vector in sub-Saharan Africa, this may have important implications for VBD modelling.

## Supplementary Files

Supplementary File 1

## Abbreviations

AIC: Akaike Information Criterion
*An. gambiae s.s.*: *Anopheles gambiae sensu stricto*
BIC: Bayesian Information Criterion
CI: Credible Interval
DD: Degree-day
EMAC: ECHAM5/MESSy2 Atmospheric Chemistry
IRS: Indoor Residual Spraying
LSM: Larval Source Management
MAP: maximum *a posteriori*
MLE: Maximum Likelihood Estimation
MTD: Minimum Temperature for Development
ODE: Ordinary Differential Equation
RH: Relative Humidity
sst: sea-surface temperature
sic: sea-ice coverage
VBD: Vector-Borne Disease
°C: degrees Celsius
