# Supplementary material for: Modelling Anopheles gambiae s.s. Population Dynamics with Temperature- and Age-Dependent Survival
Source: Int J Environ Res Public Health. 2015 May 28;12(6):5975–6005. doi: 10.3390/ijerph120605975 (PMC4483682; doi:10.3390/ijerph120605975)
Supplement: Supplementary File 1 [file ijerph-12-05975-s001.pdf]

Modelling *Anopheles gambiae s.s.* Population Dynamics with Temperature- and Age-Dependent Survival

Table S1. Comparison of the MLE-defined best-fit gamma distribution to the larval mortality data at fixed values of α. The fixed-value fit closest to the best-fit is indicated by *.

Temperature	Value of α		Difference in AIC between Best-Fit and Fixed-Alpha Value
23 °C	MLE-determined	7.27	–
	Fixed	5	22.06
		6	6.06
		7	0.24 *
		8	1.62
		9	8.32
		10	19.08
		11	33.06
		12	49.61
27 °C	MLE-determined	5.64	–
	Fixed	5	1.46
		6	0.41 *
		7	5.17
		8	13.95
		9	25.65
		10	39.57
		11	55.2
		12	72.19
31 °C	MLE-determined	7.11	–
	Fixed	5	18.09
		6	4.4
		7	0.04 *
		8	2.25
		9	9.32
		10	20.08
		11	33.74
		12	49.74
35 °C	MLE-determined	11.11	–
	Fixed	5	210.29
		6	130.67
		7	76.24
		8	39.81
		9	16.86
		10	4.32
		11	0.04 *
		12	2.5

Table S2. Comparison of the MLE-defined best fit gamma distribution to adult mortality data, with the fits given a fixed value of α. The fixed-value fit closest to the best fit is indicated by *.

Temperature	Value of α		Difference in AIC between Best Fit and Fixed-Alpha Fit
23 °C	MLE-determined	4.675	–
	Fixed	1	90.85
		2	32.05
		3	9.5
		4	1.25
		5 *	0.24
27 °C	MLE-determined	3.401	–
	Fixed	1	137.87
		2	30.42
		3 *	1.86
		4	3.33
		5	19.89
31 °C	MLE-determined	1.844	–
	Fixed	1	40.01
		2 *	0.86
		3	34.13
		4	93.67
		5	n/a

Table S3. Model sensitivity to changes in inferred parameter values.

Model	Relative Change in Likelihood Given a Relative Change in Parameter					
	n_F	q	μ_E	μ_C	τ	ΔT
1	0.026	98.191	45.755	0.029	0.029	10.287
2	0.0003	387.112	32.421	<0.001	0.31	65.249
3	23.901	175.956	56.865	40.782	0.004	49.933
4	1.003	495.651	84.885	3.176	0.002	197.08

© 2015 by the authors; licensee MDPI, Basel, Switzerland. This article is an open access article distributed under the terms and conditions of the Creative Commons Attribution license (http://creativecommons.org/licenses/by/4.0/).
